# Plasma-Enhanced Atomic Layer Deposition of AlF_3_ Antireflective Coatings via Pulse-Time Control of Fluorine Radical Reactions

**DOI:** 10.3390/nano16010043

**Published:** 2025-12-29

**Authors:** Jing Zhang, Zhixuan Zhang, Chia-Hsun Hsu, Peng Gao, Yu Qiu, Yuqi Lin, Shui-Yang Lien

**Affiliations:** 1 Xiamen Key Laboratory of Development and Application for Advanced Semiconductor Coating Technology, The School of Opto-Electronic and Communication Engineering, Xiamen University of Technology, Xiamen 361024, China; 2322111025@s.xmut.edu.cn (J.Z.); 2422111017@s.xmut.edu.cn (Y.L.); 2 Institute of Optoelectronic Display, National & Local United Engineering Lab of Flat Panel Display Technology, Fuzhou University, Fuzhou 350002, China; 241116005@fzu.edu.cn; 3 School of Advanced Manufacturing, Fuzhou University, Quanzhou 362200, China; 4 CAS Key Laboratory of Design a Assembly of Functional Nanostructures, Fujian Provincial Key Laboratory of Nanomaterials, Fujian Institute of Research on the Structure of Matter, Chinese Academy of Sciences, Fuzhou 350002, China; peng.gao@fjirsm.ac.cn; 5 Key Laboratory of Green Perovskites Application of Fujian Province Universities, Fujian Jiangxia University, Fuzhou 350108, China; yuqiu@fjjxu.edu.cn

**Keywords:** aluminum fluoride, plasma enhanced atomic layer deposition, antireflective, optical electronics

## Abstract

Plasma-enhanced atomic layer deposition (PEALD) is used to grow high-quality aluminum fluoride (AlF_3_) antireflective coatings via a safe, HF-free route using trimethylaluminum and SF_6_ plasma. In situ diagnostics reveal a reaction pathway mediated by a hydrogen-terminated fluorinated surface (s-FH). By systematically varying the plasma pulse duration, a critical process window is identified that balances efficient ligand removal against ion-induced structural damage. Within this optimized window, the films achieve ultra-low impurity levels and an atomically smooth morphology, increasing the optical transmittance of glass to (97.6 ± 0.5)%. This study establishes a clear link between fundamental plasma kinetics and functional optical performance, providing a robust, non-corrosive strategy for the rational design of metal–fluoride PEALD coatings.

## 1. Introduction

Aluminum fluoride (AlF_3_) is a compelling dielectric material, distinguished by its ultra-wide bandgap (>10 eV), very low refractive index (~1.3–1.4), and excellent chemical and thermal stability [1,2,3]. These attributes make it a key enabling material for a variety of advanced technologies, including high-performance antireflective coatings for deep-ultraviolet optics, gate dielectrics in wide-bandgap electronics, and protective layers for next-generation lithium-ion batteries [4,5,6,7,8]. Realizing these applications, however, demands AlF_3_ thin films that are not only stoichiometric and highly pure but also conformal and atomically smooth—requirements that are difficult to meet using conventional deposition techniques. Traditional approaches such as physical vapor deposition and chemical vapor deposition (CVD) often exhibit limited conformality, require elevated processing temperatures, or introduce plasma-induced damage, all of which can degrade film quality [9,10]. In contrast, atomic layer deposition (ALD) has emerged as a superior technique, offering angstrom-level thickness control, excellent conformality, and low-temperature processing capabilities [11,12,13]. The most widely used thermal ALD (TALD) route for AlF_3_ employs trimethylaluminum (TMA) and hydrogen fluoride (HF) as the precursor pair [14]. Although effective, this process relies on highly corrosive and hazardous HF, which raises serious concerns regarding safety, tool compatibility, and long-term process integration.

Plasma-enhanced ALD (PEALD) offers a promising alternative by replacing corrosive HF with reactive plasma species, such as SF_6_. Pioneering works have successfully established the baseline feasibility of this process. For instance, Vos et al. [15] first demonstrated the growth of low-refractive-index AlF_3_ films using TMA and SF_6_ plasma, proving that high-purity films could be obtained at temperatures as low as 100 °C. Building on this foundation, Messina et al. [12] provided a critical comparison between thermal and plasma-enhanced routes, highlighting that PEALD yields films with superior density and stability, particularly in the vacuum ultraviolet regime. More recently, Nieminen et al. [16] offered significant mechanistic insights by utilizing in situ diagnostics to identify the primary reaction byproducts (e.g., HF, CH_4_) and establishing the general surface reaction pathway. Huang et al. [17] further extended the application scope by integrating PEALD AlF_3_ into bilayer antireflection stacks.

Despite these significant advances, a critical knowledge gap remains regarding the kinetic control of the plasma fluorination step. Previous studies have predominantly treated the plasma exposure as a fixed parameter or focused on identifying steady-state reaction species. However, the dynamic evolution of surface reactions during the plasma pulse has not been systematically elucidated. Current literature has not fully explored the delicate competition between fluorination saturation, impurity ligand removal, and potential plasma-induced etching. While the plasma pulse time governs both the total radical dose and ion bombardment energy, its precise impact on defect formation and refractive index evolution is not yet fully understood. Without mapping these temporal dynamics, the process cannot be rationally optimized to balance film stoichiometry against plasma damage.

In this study, we address this challenge with the clear scientific objective of elucidating the time-dependent surface kinetics of SF_6_ plasma fluorination and establishing its direct correlation with film functional properties. Our central hypothesis is that the plasma step operates within a competitive kinetic regime, where a specific time window exists that is sufficient to fully eliminate organic ligands but short enough to prevent ion-induced damage. To validate this hypothesis, we employ time-resolved in situ optical emission spectroscopy (OES) and residual gas analysis (RGA) to monitor the saturation dynamics of the fluorination half-cycle. We then explicitly correlate the plasma pulse duration with impurity incorporation and refractive index evolution. Based on these insights, a refined mechanistic model is developed to rationalize the trade-off between complete fluorination and plasma-induced degradation. Finally, we demonstrate the practical utility of this mechanistic understanding by fabricating a high-performance antireflective coating, thereby linking fundamental process control to device-level optimization.

## 2. Experimental Methods

Glass substrates (2 cm × 2 cm) were cleaned using ionized water, isopropanol alcohol, and ethanol for 15 min. The cleaned substrates were then dried in an oven at 75 °C for 30 min. Afterwards, the substrates were sent to the chamber of an PEALD system (ICP-ALD 200, Xiamen Xinyifang Technology Co., Ltd., Xiamen, China). The chamber of the PEALD system was pumped down with a turbo pump to a base pressure of 3.2 × 10^−6^ Torr. A remote inductively coupled plasma (ICP) configuration was employed. The substrate holder was positioned downstream from the plasma source with a fixed plasma–substrate distance of approximately 25 cm. The ICP quartz tube was intentionally coated with polytetrafluoroethylene (PTFE) in order to resist plasma bombarding, which can eject oxygen atoms out from the quartz tube to cause oxygen contamination. The PEALD AlF_3_ films were deposited using TMA (purity: 99.999%, Aimou Yuan, Nanjing, China) precursor and SF_6_ plasma. The TMA liquid was stored in a bubbler at 25 °C, and the TMA vapor molecules were carried out from the bubbler by using nitrogen carrier gas (purity: 99.999%) of 100 sccm. The SF_6_ plasma was generated by the remote ICP source operating at 13.56 MHz with an RF power of 600 W. During the deposition process, the chamber pressure was maintained at 2.1 × 10^−1^ Torr. The SF_6_ pulse time (t) was varied from 1 to 9 s to investigate its effect on film properties. The detailed deposition parameters are summarized in Table 1. The purge gas used for TMA and SF_6_ plasma pulses was the nitrogen. The ALD cycle number was kept to 900 cycles. Due to the variation in growth kinetics, the resulting film thicknesses for the different plasma pulse times were approximately 80 nm (1 s), 110 nm (3 s), 100 nm (5 s), 75 nm (7 s), and 65 nm (9 s). These specific film thicknesses were consistent across all characterization methods employed in this study.

The thickness and refractive index of the AlF_3_ films were determined by spectroscopic ellipsometry (M2000, J.A. Woollam Co., Inc., Lincoln, NE, USA) in the spectral range of 300–800 nm at an incident angle of 65°. Data analysis was performed using the CompleteEASE software (available online: https://www.jawoollam.com/; accessed on 26 October 2025). The optical system was modeled as a four-layer stack consisting of the ambient air, a surface roughness layer, the bulk AlF_3_ film, and the glass substrate. The optical constants of the bare glass substrate were determined independently prior to film deposition to ensure modeling accuracy. The AlF_3_ layer was modeled using the Cauchy dispersion relation, where the extinction coefficient was fixed at zero. This assumption was justified by the ultra-wide bandgap of AlF_3_ which ensures negligible absorption in the visible region. Testing with non-zero extinction coefficients yielded negligible values without improving the fit quality and confirmed the validity of the transparent model. The 50% void fraction was adopted to eliminate parameter correlation between the void percentage and the roughness layer thickness. Varying this void fraction between 40% and 60% primarily influenced the calculated roughness layer thickness but resulted in negligible changes to the extracted bulk refractive index and film thickness. The OES were collected using a spectrometer (C210-DUVN, Xiamen Xinyifang Technology, Xiamen, China) over a wavelength range of 200–1000 nm, with an integration time of 100 ms and averaged over 10 acquisitions for each condition. The gas-phase species in the chamber were monitored by an RGA system (Split Flow 80, INFICON, Bad Ragaz, Switzerland) operating with an electron energy of 70 eV. The scan covered a mass-to-charge ratio (*m*/*z*) range of 1–100 amu with a step size of 0.5 amu and a dwell time of 100 ms per amu. The transmittance and reflectance spectra were measured using a UV-visible spectrometer (Lambda 850, PerkinElmer, Waltham, MA, USA). The element composition and chemical states were investigated using X-ray photoelectron spectroscopy (XPS, ESCALAB 107250Xi, Thermo Fisher Scientific, Waltham, MA, USA) using a monochromatic Al Kα radiation source (spot size: 400 µm). Survey scans were acquired with a pass energy of 150 eV and a step size of 1 eV to identify elemental presence. High-resolution core-level spectra were subsequently recorded with a pass energy of 30 eV and a step size of 0.1 eV to resolve detailed chemical bonding states. Prior to the XPS measurements, the surface of the samples was etched by an Ar+ ion beam. The surface morphology and root-mean-square surface roughness were obtained using an atomic force microscopy (AFM, XE7, Park-Systems, Suwon, Republic of Korea). The surface morphology and roughness were characterized by atomic force microscopy (AFM, XE7, Park-Systems, Suwon, Korea) in tapping mode using a silicon cantilever (tip radius < 10 nm). Height images were typically recorded over a 5 × 5 μm^2^ area with a resolution of 512 × 512 pixels at a scan rate of 1 Hz. The root-mean-square (RMS) roughness was extracted from the height maps after standard plane correction. The crystalline structure of the films was evaluated by grazing-incidence X-ray diffraction (GIXRD, TTRAXIII, Rigaku, Japan) using Cu Kα radiation (λ = 0.15418 nm). The measurements were conducted at a fixed incidence angle of 0.5°, with patterns collected over a 2θ range of 10–80° at a scan rate of 1°/min (step size: 0.02°). To ensure the reproducibility of the results, three independent deposition runs were performed for each experimental condition on different days. Consequently, all quantitative data reported herein for growth per cycle (GPC), F/Al atomic concentration ratios, RMS roughness, and optical transmittance are presented as the mean with standard deviation.

## 3. Results and Discussion

**Figure 1:**
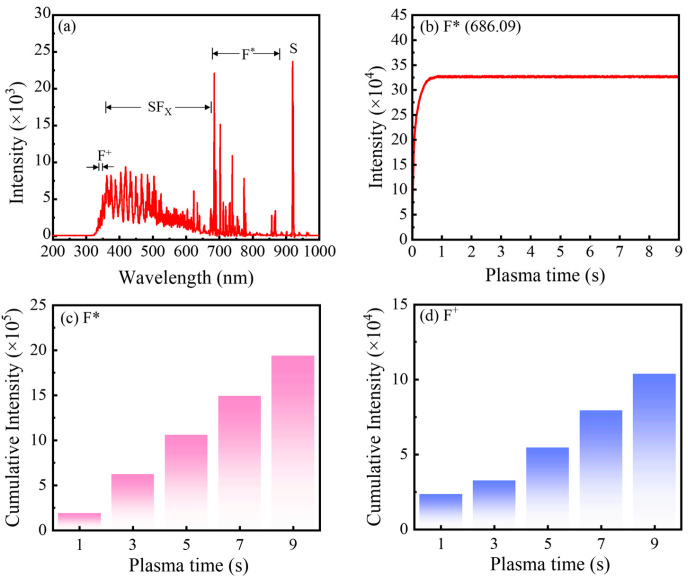
(**a**) OES of SF_6_ plasma at 600 W. Temporal evolution of (**b**) F* peak intensity, and accumulative intensity of (**c**) F* and (**d**) F^+^ as a function of plasma pulse time. The substrate temperature was 200 °C, and the chamber pressure was 2.1 × 10^−1^ Torr.

Figure 1a shows the optical emission spectra (OES) of the SF_6_ plasma used in this work. Under plasma discharge, SF_6_ molecules are dissociated by electron-impact collisions, and a fraction of the resulting fragments are excited to higher electronic states. Their subsequent relaxation to lower energy levels emits photons that are captured by OES. The broad features observed at 350–670, 670–870, and 921 nm are attributed to SF_x_ (1 ≤ x ≤ 5), F*, and S species, respectively [16,18]. In addition, the peaks at 340–350 nm are assigned to F^+^ species, as confirmed by comparison with the NIST database [19]. To assess the temporal stability of the SF_6_ plasma during the pulse, the intensity of the most prominent fluorine emission line (F* at 686.09 nm) was monitored as a function of SF_6_ plasma pulse time. As shown in Figure 1b, the F* intensity increases initially and then reaches a plateau, remaining essentially constant from 1 s to 9 s. This behavior directly evidences a stable and reproducible plasma discharge over this time window, implying a well-controlled and repeatable flux of reactive species impinging on the substrate during PEALD. Figure 1c,d present the integrated emission intensities of F* (670–870 nm) and F^+^ (340–350 nm) as a function of SF_6_ plasma pulse time. Both species exhibit a nearly linear increase in accumulated intensity with increasing pulse time, reflecting the progressively larger total dose of fluorine-containing species delivered to the surface. The neutral fluorine radicals (F*) are particularly critical, as they react with the TMA-derived methyl-terminated surface to efficiently remove CH_3_ ligands and form Al–F bonds, thereby enabling self-limiting fluorination. In contrast, although a modest concentration of F^+^ has limited impact on film growth, an excessive ion flux can strongly bombard the growing surface, break bonds, and introduce ion-induced damage. This ultimately degrades film quality, underscoring the need to carefully balance neutral radical supply against ion bombardment when optimizing the plasma pulse time for high-quality AlF_3_ PEALD films.

**Figure 2:**
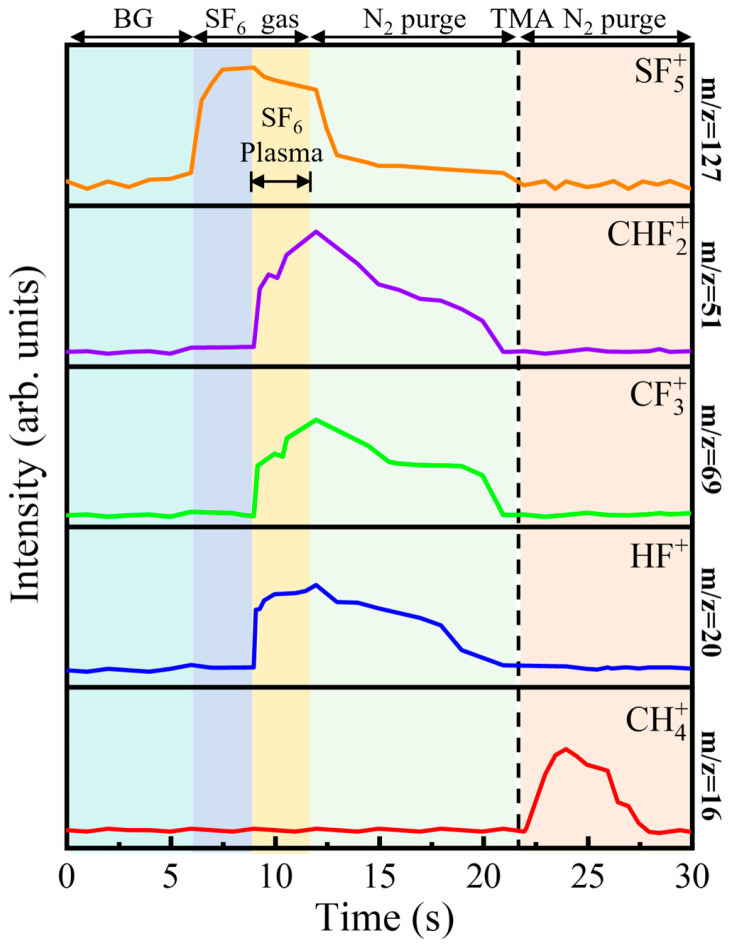
RGA signals of the background (BG) and the two half-reactions with a 3 s SF_6_ plasma pulse time. The substrate temperature was 200 °C, and the chamber pressure was 2.1 × 10^−1^ Torr.

Figure 2 presents the temporal evolution of key mass-to-charge (*m*/*z*) signals, monitored by in situ RGA, thereby resolving the sequence of chemical events within a single PEALD cycle. At the beginning of the cycle, all tracked signals remain at a stable, low-intensity background level, indicating an inert gas-phase environment. The first half-reaction is initiated at 6 s by igniting the SF_6_ plasma. The sharp rise in the signal at *m*/*z* = 127, attributed to SF_5_^+^—the principal fragment of SF_6_ [16,20,21]—confirms effective precursor dissociation and the rapid generation of fluorine-containing plasma species. Simultaneously, a series of reaction byproducts emerges: carbon tetrafluoride (CF_4_, identified via its dominant fragment CF_3_^+^ at *m*/*z* = 69 [16]), trifluoromethane (CHF_3_, via CF_2_H^+^ at *m*/*z* = 51 [16]), and hydrogen fluoride (HF, *m*/*z* = 20 [14,16,22]). The concurrent appearance of CF_4_ and CHF_3_ is particularly revealing. It indicates that the removal of surface methyl groups proceeds via two parallel fluorination pathways. The formation of CF_4_ corresponds to a complete fluorination route, in which all C–H bonds are replaced by C–F bonds under the action of the highly reactive, fluorine-rich plasma. In contrast, the formation of CHF_3_ reflects a reaction pathway yielding a volatile species in which one C–H bond remains intact. It is crucial to note that both CF_4_ and CHF_3_ serve as effective elimination routes for removing carbon from the surface. Analysis of the RGA signal intensities reveals that these two pathways operate in parallel with a relatively constant ratio throughout the plasma pulse (1–9 s). This stability confirms that the reaction mechanism itself does not change over time. The completeness of ligand removal is strictly governed by the cumulative reaction time. After the RF power is switched off at 9 s, a 10 s purge step efficiently removes the gaseous reactants and byproducts, as evidenced by all mass signals returning to their initial background levels. The second half-reaction begins at 18 s with the introduction of the TMA pulse. This step is marked by a pronounced increase in the signal at *m*/*z* = 16, corresponding to CH_4_, which is observed as the sole volatile byproduct [15,16,23]. The exclusive formation of methane is a crucial mechanistic signature: it implies that the preceding plasma step not only strips the methyl ligands but also leaves the surface terminated with hydrogen-containing fluorinated species. These surface—F(H) groups then react cleanly with incoming TMA to produce CH_4_, consistent with a well-defined, self-limiting ligand-exchange reaction. Together, the RGA data in Figure 2 provide a coherent, time-resolved picture of the PEALD cycle, directly linking plasma-driven fluorination chemistry to the formation of a protonated fluorine–terminated surface that underpins the subsequent AlF_3_ growth.

**Figure 3:**
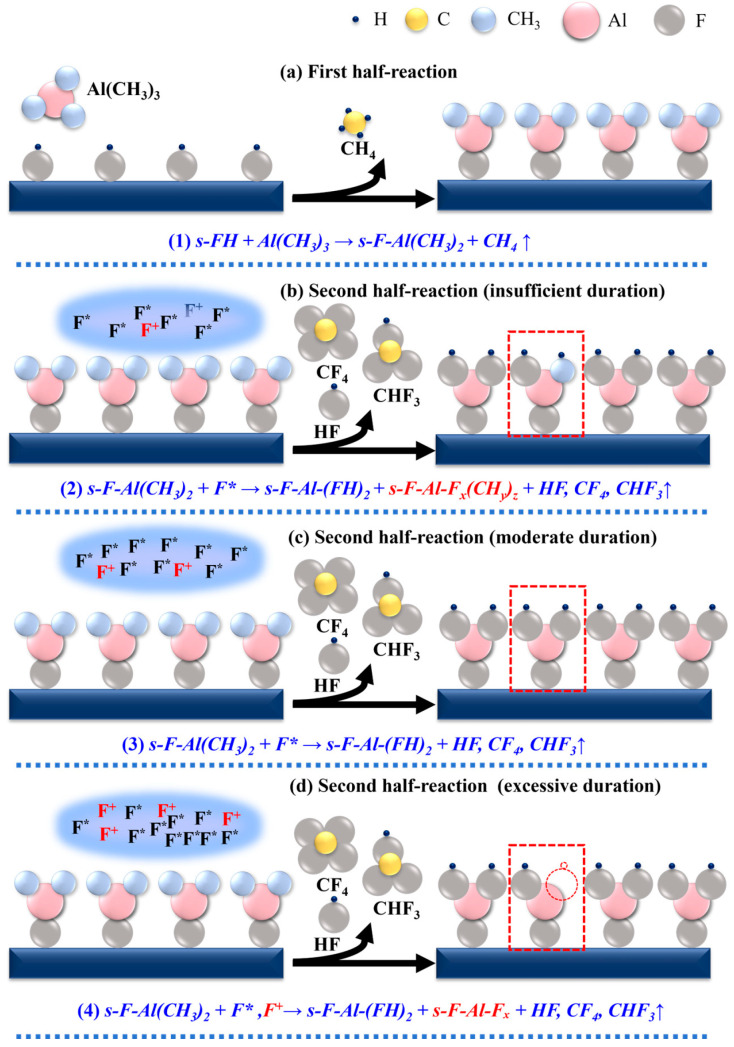
Schematic diagram of the PEALD mechanism of AlF_3_ films.

Based on the in situ OES and RGA results, a detailed surface reaction mechanism for the PEALD of AlF_3_ is proposed, as schematically illustrated in Figure 3. In the absence of direct vibrational spectroscopy, the RGA data indicates that the deposition proceeds on a hydrogen-terminated fluorinated surface (tentatively s-FH). This is supported by the consistent detection of CH_4_ during the TMA half-cycles. Residual OH groups or hydrogen on the glass substrate could theoretically generate CH_4_, but this influence is restricted to the very first few ALD cycles. Once the growing AlF_3_ film fully covers the substrate, the CH_4_ generation observed during steady-state growth implies that FH ligands are continuously regenerated on the film surface during the SF_6_ plasma step. This regeneration mechanism aligns with prior reports on ALD AlF_3_, where such a termination is considered essential for the reaction with TMA [14,15]. On this basis, the self-limiting TMA half-reaction can be plausibly written as:

(1) s-FH + Al(CH_3_)_3_ → s-F-Al(CH_3_)_2_ + CH_4_

where s denotes the substrate surface. After the TMA pulse and subsequent purge, residual gas-phase species and CH_4_ are removed, leaving the surface terminated with Al(CH_3_)_2_ groups. The second half-reaction involves exposing this Al(CH_3_)_2_-terminated surface to the SF_6_ plasma. The completeness of this step—and thus the resulting film quality—depends critically on the plasma pulse time, leading to three distinct regimes, as depicted in Figure 3. (i) Short plasma exposure (t = 1 s). For a short SF_6_ plasma pulse, the total dose of fluorine radicals and energetic ions is insufficient to drive ligand exchange to completion across the entire surface. As a result, the removal of carbon-containing ligands is incomplete, yielding a mixed surface that contains fully converted AlF_3_ alongside residual carbonaceous fragments. This can be summarized as:

(2) s-F-Al(CH_3_)_2_ + F* → s-F-Al-(FH)_2_ + s-F-Al-F_x_(CH_y_)_z_ + HF, CF_4_, CHF_3_

where F* denotes neutral fluorine radicals. In this regime, the process must also regenerate the active s-FH sites required for the next cycle. This regeneration is likely driven by surface protonation facilitated by the abundant HF generated in situ, as suggested by the HF RGA signal. (ii) Self-limiting PEALD window (3 ≤ t < 7 s). When the plasma pulse time is sufficiently long (e.g., 3–5 s), the F* dose becomes adequate to fully remove the methyl ligands and to completely fluorinate the surface, forming a dense, stoichiometric AlF_3_ layer. This regime is consistent with the simultaneous detection of CF_4_ (via its *m*/*z* = 69 fragment) and CHF_3_ (*m*/*z* = 51), reflecting the coexistence of two parallel volatile pathways (CF_4_ and CHF_3_) that together ensure exhaustive ligand removal. The overall reaction can be written as:

(3) s-F-Al(CH_3_)_2_ + F* → s-F-Al-(FH)_2_ + HF, CF_4_, CHF_3_

Within this window, minor variations with pulse time still exist. For example, as the exposure is extended from 3 s to 5 s, a slightly higher ion flux may begin to introduce a small degree of structural disorder. Nevertheless, the films remain chemically pure and highly dense, corresponding to the optimal growth regime identified experimentally. (iii) Excessive plasma exposure (7 ≤ t ≤ 9 s). For longer plasma pulses, the surface is subjected to substantial bombardment by a high flux of F^+^ ions, while the chemical fluorination reaction is already complete. In this overexposed regime, the dominant effect shifts from chemistry to ion-driven physics: sustained ion bombardment can induce bond breaking, generate defects, increase surface roughness, and reduce film density through sputtering or microstructural damage. This can be conceptually expressed as:

(4) s-F-Al(CH_3_)_2_ + F*, F+ → s-F-Al-(FH)_2_ + s-F-Al-F_x_ + HF, CF_4_, CHF_3_

where s-F-Al-F_x_ denotes a damaged, sub-stoichiometric Al-F_x_ environment. Although direct ion flux measurements were not performed, the transition to an ion-driven regime is rationalized by the specific configuration of the remote ICP source. Based on literature for similar remote ICP reactors, the average ion energy is expected to be comparable to 9 ± 1 eV, with an ion flux of approximately 10^13^ cm^−2^s^−1^ [24]. Consequently, the critical factor is not the single-particle energy but rather the cumulative ion-energy dose defined as the product of mean energy, flux, and time. As evidenced by the linear rise in F^+^ emission intensity in Figure 1d, the total energy delivered to the surface increases continuously with pulse duration. In this work, the fluorination reaction reaches chemical saturation at 3–5 s. Beyond this saturation point, the sustained bombardment at 7–9 s provides a high cumulative dose that disrupts the Al-F bonding equilibrium. This promotes the preferential sputtering (or selective stimulated desorption) of the lighter fluorine atoms, which are more susceptible to energy-induced removal than Al atoms. This shift from self-limiting growth to energy-induced fluorine loss accounts for the decline in growth rate, the fluorine deficiency, and the increased surface roughness observed in the following GPC, XPS, and AFM characterizations. The fluorine deficiency corresponds to the generation of point defects, specifically anion vacancies or unsaturated metal sites within the lattice. Comparative analysis with well-studied fluorides offers critical insights into the potential consequences of these defects. For instance, Romanova et al. [25] demonstrated that defect sites in CaF_2_ serve as charge traps that fundamentally alter dielectric behavior. Similarly, Skriabin et al. [26] showed that radiation-induced surface defects in MgF_2_ can facilitate oxygen diffusion, thereby accelerating surface degradation and oxidation. Drawing from these findings, it can be predicted that the F-deficient PEALD AlF_3_ films generated in the excessive exposed regime are likely susceptible to similar failure modes, where anion vacancies could act as charge traps or pathways for post-deposition oxidation, ultimately compromising optical stability. Taken together, the mechanism reveals that an optimal plasma pulse time must balance two competing requirements: supplying sufficient F* radicals to ensure complete ligand removal and full fluorination, while avoiding excessive F^+^ bombardment that compromises structural integrity. This framework provides a clear mechanistic basis for the subsequent correlation between plasma pulse time, film composition and microstructure, and ultimately the optical performance of the AlF_3_ coatings. To investigate the influence of SF_6_ plasma pulse time on the AlF_3_ deposition, the growth characteristics are studied.

**Figure 4:**
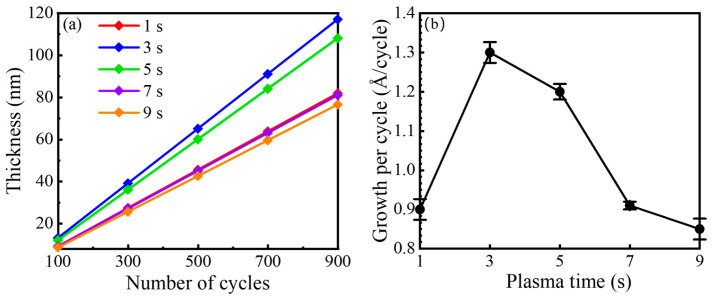
(**a**) AlF_3_ film thickness as a function of ALD cycle number for different SF_6_ plasma pulse times. (**b**) GPC as a function of plasma pulse time.

Figure 4a displays the film thickness as a function of the number of PEALD cycles for various plasma pulse times. For all conditions, the film thickness exhibits a good linear dependence on the number of cycles. This linearity confirms that each cycle contributes a consistent and repeatable amount of material, which is a hallmark of a self-limiting deposition process [13,27]. To rigorously validate the ideal ALD characteristics of the developed process, the saturation behavior of the precursor half-reaction and the temperature dependence are evaluated, as detailed in Figure S1 (Supporting Information). The GPC remains constant for TMA pulse times exceeding 0.1 s, confirming that the precursor adsorption is self-limiting. Furthermore, the growth rate exhibits a stable plateau across a substrate temperature range of 200–300 °C, establishing an ALD temperature window. These results, combined with the strict linear relationship between film thickness and cycle number shown in Figure 4a, unequivocally classify the process as true ALD. The GPC was extracted from the slope of these linear fits, and the results are plotted against the plasma pulse time in Figure 4b. The GPC plot provides compelling quantitative evidence for the three reaction regimes hypothesized previously. At t = 1 s, the GPC is suppressed to 0.91 ± 0.03 Å/cycle. This confirms incomplete surface reactions and thus a reduced growth rate, as postulated. As t = 3 and 5 s, the GPC rises to a stable plateau of approximately (1.20 ± 0.02)–(1.30 ± 0.02) Å/cycle. This distinct saturation behavior defines the ALD window, in which the plasma dose is sufficient for complete chemical reactions. For longer exposures of t = 7 and 9 s, a clear decrease in GPC to (0.90 ± 0.02) and (0.85 ± 0.02)Å/cycle, respectively, is observed. This trend strongly supports the existence of a competing soft-etching or sputtering effect caused by prolonged ion bombardment, which becomes significant after chemical saturation is achieved.

**Figure 5:**
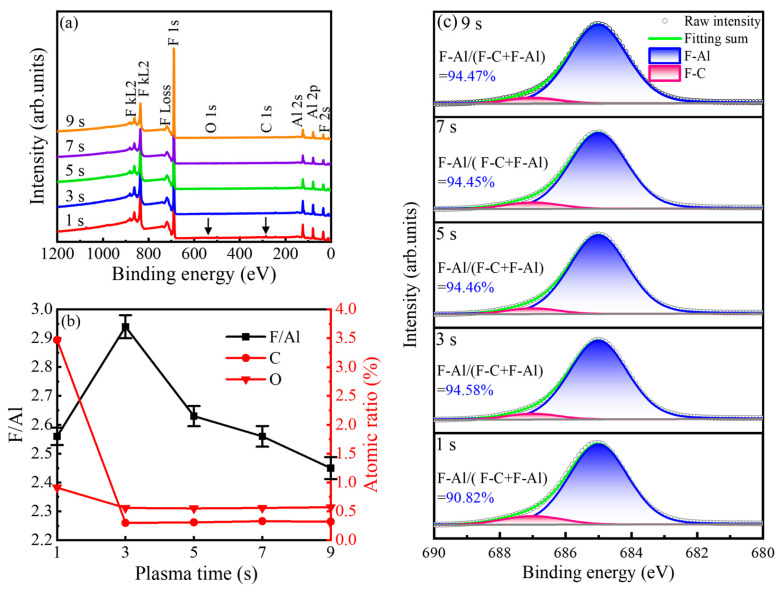
(**a**) XPS survey spectra, (**b**) F/Al elemental ratio and impurity content, (**c**) high-resolution F 1s spectra for the films prepared at different plasma pulse times.

In Figure 5a, the XPS survey spectra for AlF_3_ films grown with plasma pulse times between 1 and 9 s are dominated by intense Al 2p/2s and F 1s/F KLL features, while C 1s and O 1s peaks remain barely above the detection limit. This immediately points to a highly fluorinated aluminum matrix with only trace levels of extrinsic impurities. Quantitative analysis of the high-resolution spectra, summarized in Figure 5b, shows that the F/Al atomic ratio follows a pronounced non-monotonic dependence on plasma pulse time. Starting from a sub-stoichiometric value of 2.55 ± 0.03 at 1 s, the ratio increases to a maximum of 2.94 ± 0.04 at 3 s. The small standard deviation derived from three independent runs demonstrates high process reproducibility. Considering the typical XPS quantification uncertainty of ~10% [28], the measured value is experimentally indistinguishable from the ideal stoichiometric ratio of 3.00. This confirms the formation of fully fluorinated AlF_3_. The ratio then gradually decreases to 2.48 ± 0.04 at 9 s. This evolution closely tracks the GPC behavior: the initially low ratio reflects incomplete fluorination under insufficient plasma exposure, the peak at 3 s marks the condition of most efficient fluorine incorporation within the self-limiting PEALD window, and the subsequent decline at longer pulses is consistent with preferential sputtering of the lighter fluorine atoms during prolonged ion bombardment, yielding an increasingly Al-rich film. For the impurity profiles, carbon serving as a direct probe of TMA ligand removal is strongly elevated at short plasma exposure: at 1 s, the carbon content reaches 3.47 at.%, indicating a substantial population of residual carbonaceous fragments. This high impurity level aligns with the kinetic limitation suggested by the RGA data. Since the CF_4_ and CHF_3_ removal routes operate at a constant ratio, the residual carbon at short pulse times is not due to a shift in reaction pathway, but because the short plasma duration is insufficient to convert all surface methyl ligands into these volatile byproducts. When the plasma pulse is extended to 3 s, the carbon concentration collapses to ~0.3 at.% and remains at this low level for all longer exposures, demonstrating that a 3 s plasma step is sufficient to fully strip the organic ligands. Oxygen levels are consistently below 0.56 at.% across all conditions, underscoring the robustness of the oxygen-free PEALD chemistry. It is noted that hydrogen cannot be quantified by XPS, and since complementary techniques such as elastic recoil detection analysis or secondary ion mass spectrometry are not performed, the specific hydrogen content in these films remains an assumption that is not directly verified in this study. Nevertheless, the presence of hydrogen is chemically deduced from the RGA data, where the exclusive formation of methane necessitates a hydrogen source on the surface. Consistent with this observation, literature on similar PEALD AlF_3_ processes explicitly reports hydrogen concentrations of ~1.7 at.% [15], suggesting that a comparable trace incorporation is likely present in our films. The evolution of fluorine bonding is further clarified in Figure 5c, where the F 1s core-level spectra are deconvoluted into two components: a dominant peak at ~685.5 eV attributed to F-Al bonds and a weaker shoulder at ~687.0 eV assigned to F–C species [10,27]. The fraction of the F-Al component in the total F 1s signal mirrors the trends derived from the elemental analysis. At 1 s, the reduced F-Al fraction reflects incomplete surface reactions and a significant contribution from residual carbonaceous species, consistent with the mechanism described by Equation (2). Under the optimal plasma exposure, the F-Al fraction increases to ~94.6%, indicating that fluorine radicals efficiently convert methyl groups into volatile CF_4_ and CHF_3_ byproducts and drive the surface toward a fully fluorinated AlF_3_ network. Collectively, the XPS results in Figure 5a–c provide chemically resolved confirmation that a 3 s plasma pulse defines the optimal PEALD window, balancing complete ligand removal and fluorination against the onset of ion-induced fluorine depletion at longer exposures.

**Figure 6:**
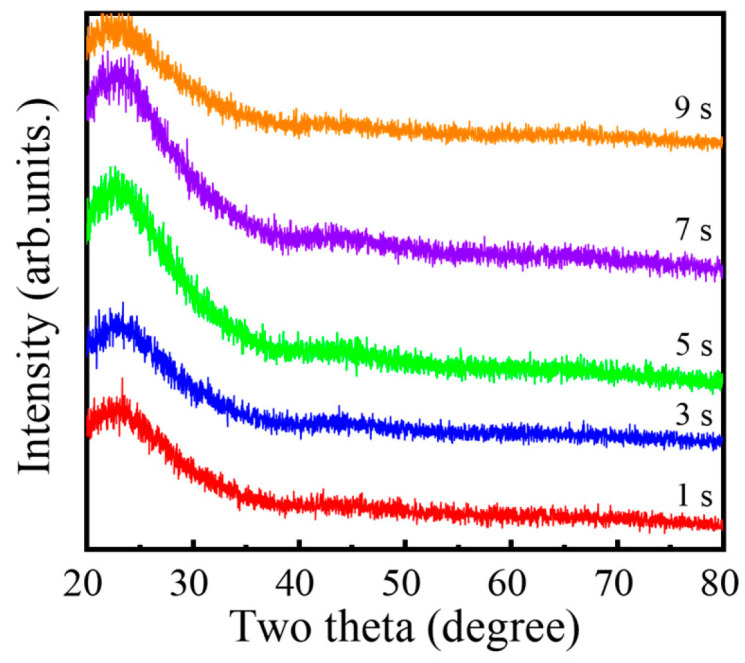
GIXRD patterns of the PEALD AlF_3_ films deposited with different plasma pulse times.

In Figure 6, the GIXRD patterns of the AlF_3_ films deposited with different plasma pulse times show no sharp Bragg reflections, only broad diffuse features, confirming that all films are amorphous. Such an amorphous structure is highly advantageous for antireflective applications because it naturally suppresses grain-boundary formation and associated surface corrugation. In contrast to polycrystalline coatings, which often exhibit pronounced grain boundaries and faceted surfaces, these amorphous AlF_3_ layers can develop an exceptionally smooth interface. The resulting reduction in surface scattering is expected to enhance optical transmittance and is therefore fully consistent with the excellent antireflection performance observed for these films [14,29].

**Figure 7:**
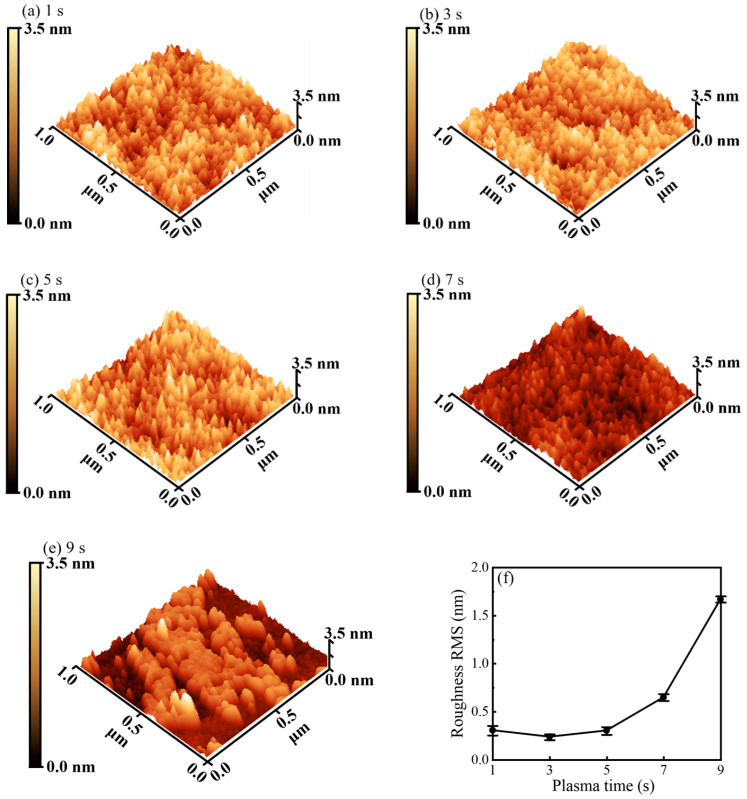
AFM topography images for the films deposited at the plasma pulse times of (**a**) 1, (**b**) 3, (**c**) 5, (**d**) 7 and (**e**) 9 s. (**f**) RMS surface roughness as a function of plasma pulse times.

In Figure 7a–e, AFM images reveal a pronounced dependence of surface morphology on plasma pulse time. Among all conditions, a 3 s plasma pulse produces the smoothest surface, whereas longer pulses (particularly 9 s) lead to visibly roughened topography. The corresponding RMS roughness values are summarized in Figure 7f. The minimum RMS roughness of 0.24 ± 0.03 nm is obtained at 3 s, coinciding with the saturation of the surface reactions. Such an exceptionally smooth surface is crucial for high-performance antireflective coatings, as it minimizes scattering losses and thus supports high optical transmittance. When the plasma exposure is further extended, the RMS roughness increases markedly, reaching 1.67 ± 0.03 nm at 9 s. This pronounced roughening is a direct morphological consequence of excessive plasma bombardment: once the chemical conversion is complete, continued ion impact effectively transitions to physical etching, damaging the film and introducing nanoscale height fluctuations. These features inherently degrade the antireflective properties by enhancing scattering and haze. The close correspondence between the reaction-saturation point (3 s) and the morphological optimum provides strong physical support for the proposed mechanistic picture and underscores the need to balance sufficient radical/ion flux against plasma-induced damage when optimizing PEALD processes for optical coatings.

**Figure 8:**
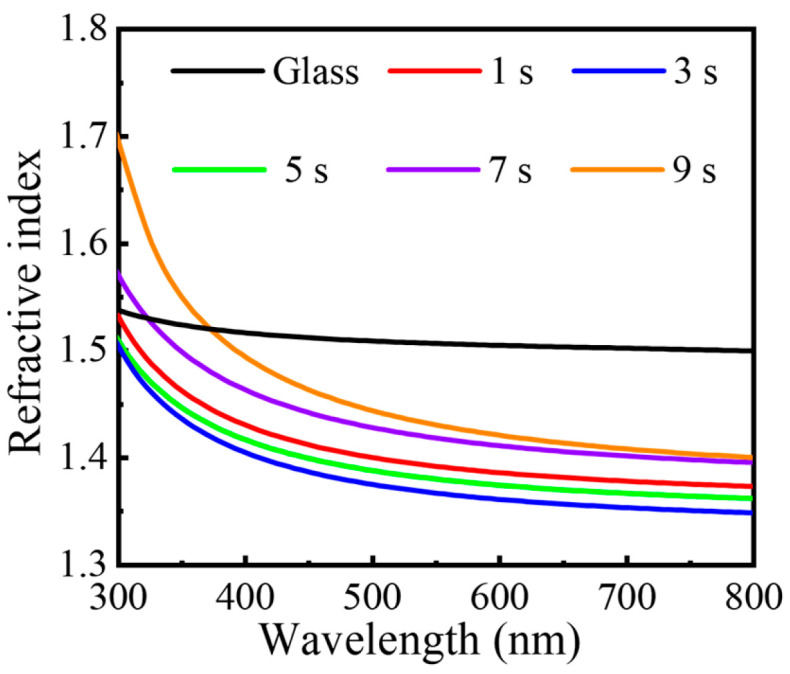
Refractive index spectra of AlF_3_ films with different plasma pulse times.

In Figure 8, the wavelength-dependent refractive index spectra of the AlF_3_ films are compared with that of bare glass, which exhibits a refractive index of ~1.50–1.53 across the visible range. The film deposited with a 3 s plasma pulse time shows the lowest refractive index over the entire spectral window, reaching values as low as 1.35 at 550–800 nm. This ultralow index is characteristic of a high-quality AlF_3_ layer and is fully consistent with the optimal composition and morphology identified above. As the plasma pulse time deviates from 3 s, the refractive index systematically increases. In particular, films grown with longer pulses (5–9 s) display progressively higher indices, which can be ascribed to plasma-induced damage and fluorine depletion, leading to a more Al-rich, higher-index network. Because a low refractive index is essential for efficient antireflection, the AlF_3_ film obtained at a 3 s plasma pulse clearly provides the most favorable optical response and is therefore selected for detailed antireflective performance evaluation.

To synthesize the experimental findings presented above, Table 2 provides a comprehensive summary of the three distinct kinetic regimes governed by the plasma pulse time. This tabulated overview explicitly links the real-time plasma diagnostics and surface reaction pathways to the resulting film properties. The optimal process window (Regime II) represents a critical balance where the plasma dose is sufficient to drive complete ligand exchange to high purity and stoichiometry while remaining moderate enough to prevent the ion-induced damage and roughening observed in the overexposed regime (Regime III).

**Figure 9:**
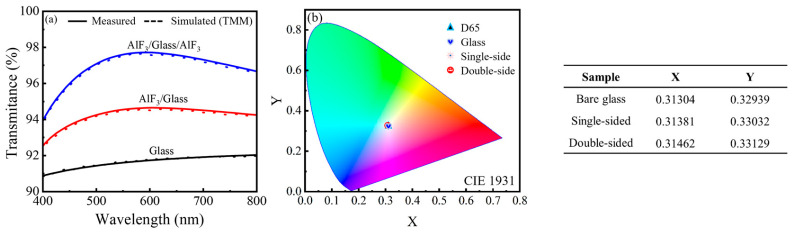
(**a**) Comparison of measured (solid lines) and simulated (dashed lines) transmittance spectra of single-sided and double-sided AlF_3_-coated glass versus bare glass. The simulation is based on the TMM model using ellipsometry-derived optical constants. (**b**) Transmission CIE 1931 (x, y) Color Coordinates of Bare and Coated Samples (D65, 2° Observer).

In Figure 9a, the antireflective performance of the optimized PEALD AlF_3_ film grown with a 3 s plasma pulse is evaluated on glass substrates with a film thickness of 110 nm. The bare glass exhibits an average transmittance of ~(91.5 ± 0.2)%, limited by reflection losses of ~4–5% at each air/glass interface, in good agreement with Fresnel’s equations [30,31,32,33]. Upon coating a single side with AlF_3_, the average transmittance increases markedly to (94.5 ± 0.4)%, directly reflecting the suppression of front-surface reflection through effective refractive-index matching. When both sides are coated, the average transmittance is further boosted to an impressive (97.6 ± 0.5)%, demonstrating that the AlF_3_ layer efficiently suppresses reflection losses across the entire visible spectrum. To verify the accuracy of the extracted optical constants and assess the theoretical limit of the coating, optical simulations based on the transfer-matrix method (TMM) were performed. The model utilized the refractive index dispersion and thickness values determined from ellipsometry, treating the AlF_3_ layer as a coherent thin film and the thick glass substrate as an incoherent medium. As shown in Figure 9a, the simulated spectra (dashed lines) overlay the measured data (solid lines) with remarkable precision. The excellent agreement between the model and the experiment confirms the validity of the optical parameters and demonstrates that the PEALD AlF_3_ films achieve an antireflective performance virtually identical to the theoretical design target. Figure 9b shows the corresponding transmission color coordinates (CIE 1931 (x, y)) for the bare and coated samples, calculated from the measured transmittance spectra using the D65 illuminant and the 2° standard observer. While the bare glass lies close to the D65 white point, both the single- and double-sided AlF_3_-coated glasses shift toward the theoretical achromatic point (x = 0.333, y = 0.333). This behavior indicates that the PEALD AlF_3_ coating not only enhances the overall transmittance but also improves the color neutrality of transmitted light, effectively reducing residual tint from the substrate. Taken together, these optical results establish the PEALD AlF_3_ film as a highly effective, color-neutral antireflective coating. The combination of ultralow refractive index, extremely smooth morphology, and excellent stoichiometric control enables simultaneous optimization of transmission efficiency and spectral neutrality—key requirements for high-end optical components, display cover glasses, and precision sensing systems.

**Figure 10:**
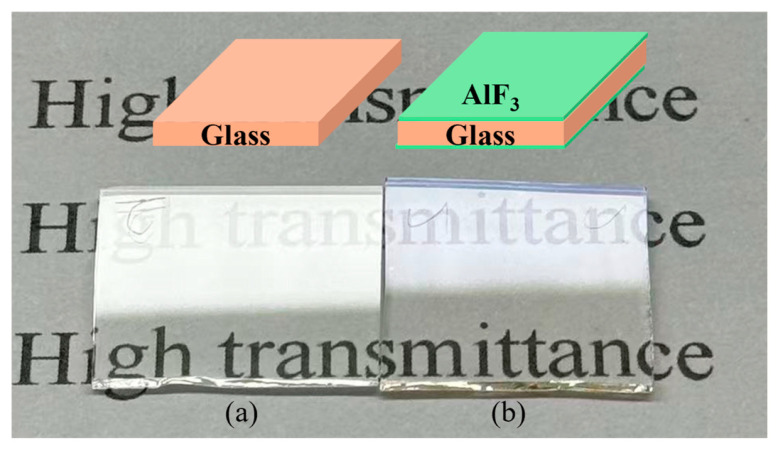
Visual comparison of transmission enhancement for (**a**) bare glass, and (**b**) double-sided AlF_3_-coated samples under fluorescent lighting.

In Figure 10, photographs of a bare glass substrate and a double-sided AlF_3_-coated sample are shown under identical fluorescent illumination positioned directly above the specimens and viewed at an oblique angle. The bare glass exhibits strong surface reflectivity, evidenced by pronounced, bright glare from the light source that partially obscures the underlying text. By contrast, the AlF_3_-coated glass shows a marked suppression of this specular reflection, rendering the printed letters beneath the substrate noticeably clearer and more legible. The reduced glare and enhanced contrast provide an intuitive, side-by-side visualization of the antireflective effect, in excellent agreement with the quantitative transmittance and colorimetric data. This simple optical demonstration highlights the practical efficacy of the PEALD AlF_3_ coating in improving visual clarity and overall light transmission in real-world viewing conditions.

Table 3 compares the AlF_3_ film properties and antireflective performance achieved in this work with those reported previously. Conventional physical vapor routes, such as evaporation and sputtering, typically produce AlF_3_ coatings with relatively high RMS roughness (≈1.45 nm and ≈0.8 nm, respectively [23,29]), which enhances light scattering and inherently limits antireflective performance. Among ALD methods, TALD processes using metal–fluoride precursors (e.g., TiF_4_ or TaF_5_) can avoid the use of HF but often suffer from significant impurity incorporation (2–8.3 at.%) or lower growth rates [14,34,35]. Similarly, ALD routes relying on anhydrous HF, including both thermal and plasma-enhanced modes, not only raise serious safety and equipment corrosion concerns but also typically yield residual impurity levels between 2 and 14 at.% [12,17,36]. Even regarding the safer SF_6_-based PEALD approach, previous studies generally reported impurity contents in the range of 2.0–2.7 at.% [15,37,38]. In distinct contrast, the PEALD strategy developed here advances the state-of-the-art by simultaneously optimizing safety, purity, and morphology. By combining high-vacuum pre-deposition with a PTFE-lined plasma tube and optimized pulse kinetics, we achieve an ultra-low total impurity content of only 0.86 at.%. This represents a significant improvement not only over hazardous HF-based methods but also over prior SF_6_-based reports. Furthermore, our process delivers an atomically smooth surface (RMS ~0.24 ± 0.03 nm) and a low refractive index (1.35), both hallmarks of dense, high-quality AlF_3_ films. Taken together, these results demonstrate that our HF-free PEALD process offers a safer, more robust route that surpasses existing methods in both structural quality and functional optical performance.

## 4. Conclusions

A mechanistically elucidated, HF-free PEALD process for AlF_3_ using TMA and SF_6_ plasma has been established. In situ diagnostics demonstrate that the plasma pulse time is the decisive parameter governing three distinct growth regimes: undersaturated growth, self-limiting saturation, and ion-induced degradation. Operating within the optimized window yields ultra-smooth, high-purity AlF_3_ films with low refractive index and superior antireflective performance. This pulse-time-controlled strategy effectively bridges the gap between process safety and film quality, achieving a combination of stoichiometric control and minimal surface scattering without relying on hazardous HF precursors. Future work will focus on evaluating the environmental stability of these coatings, particularly under humidity, thermal cycling, and UV exposure. Additionally, scaling the process to industry-relevant substrates while maintaining ultra-low roughness represents a key direction for subsequent research.:

## Figures and Tables

**Table 1:** PEALD AlF_3_ thin film preparation parameters.

Parameter	Value
TMA bubbler temperature (°C)	25
Substrate temperature (°C)	200
TMA pulse time (s)	0.1
TMA purge time (s)	6
TMA purge gas flow rate (sccm)	100
SF_6_ pulse time (s)	1–9
SF_6_ purge time (s)	10
SF_6_ flow rate (sccm)	50
SF_6_ purge gas flow rate (sccm)	100
SF_6_ plasma power (W)	600

**Table 2:** Mechanistic summary of the PEALD AlF_3_ process regimes, correlating plasma dynamics with film properties.

Regime	Dominant Mechanism	Diagnostic Signatures (OES and RGA)	Film Properties
I. Under-saturated t < 3 s	Incomplete reaction: Residual CH_3_ ligands	Low F*, F^+^; Weak CF_4_, CHF_3_, HF	High impurity; F-deficient; Low GPC
II. PEALD window 3 ≤ t < 7 s	Self-limiting saturation: Full surface fluorination	Moderate F*, F^+^; Strong CF_4_, CHF_3_, HF	Minimum impurity; Maximum GPC; Stoichiometric; Minimum RMS
III. Over-exposed 7 ≤ t ≤ 9 s	Ion damage: Preferential F sputtering; Al-F bond breaking	Excessive F*, F^+^; declining CF_4_, CHF_3_, HF	Reduced GPC; F-deficient (defects); Increased RMS

**Table 3:** Comparison of AlF_3_ film properties between this work and literature. The abbreviations of temp. and imp. stand for temperature and impurity content, respectively.

Method	Temp. (°C)	Al Source	F Source	*n* (~600 nm)	F/Al (Ratio)	Imp. (at.%)	RMS (nm)	GPC (Å/cycle)	Ref.
TALD	150	TMA	TiF_4_	~1.38	3.08	8.3	1.1	0.54	[35]
TALD	240	AlCl_3_	TiF_4_	~1.36	3.20	~3	0.6	0.80	[34]
TALD	200	TMA	TaF_5_	~1.36	~2.40	~2	-	0.40	[14]
TALD	200	TMA	HF	-	3.10	5	-	0.40	[36]
TALD	100	TMA	HF	-	3.10	2	-	1.10	[12]
PEALD	100	TMA	HF + H_2_	-	2.80	14	-	0.70	[12]
PEALD	100	TMA	HF + H_2_	1.44	-	2.4	0.49	1.30	[17]
PEALD	150	TMA	SF_6_	-	2.90	2	0.88	~1.20	[37]
PEALD	200	TMA	SF_6_	-	2.70	2	-	0.63	[38]
PEALD	200	TMA	SF_6_	~1.35	2.90	2.7	0.23	0.85	[15]
PEALD	200	TMA	SF_6_	~1.35	2.94	0.86	0.24	1.30	This work

## Data Availability

The original contributions presented in this study are included in the article/Supplementary Materials. Further inquiries can be directed to the corresponding authors.

## Supplementary Materials

The following supporting information can be downloaded at: https://www.mdpi.com/article/10.3390/nano16010043/s1, Figure S1. GPC as a function of (a) TMA pulse time and (b) substrate temperature.

## References

[1] Hennessy J., Jewell A.D., Balasubramanian K., Nikzad S. (2016). Ultraviolet optical properties of aluminum fluoride thin films deposited by atomic layer deposition. J. Vac. Sci. Technol. A Vac. Surf. Film..

[2] Christidis G., Koch U., Poloni E., Leo E.D., Cheng B., Koepfli S.M., Dorodnyy A., Bouville F., Fedoryshyn Y., Shklover V. (2020). Broadband, High-Temperature Stable Reflector for Aerospace Thermal Radiation Protection. ACS Appl. Mater. Interfaces.

[3] Siqueiros J.M., Machorro R., Regalado L.E. (1988). Determination of the optical constants of MgF_2_ and ZnS from spectrophotometric measurements and the classical oscillator method. Appl. Opt..

[4] Sun J., Li X., Zhang W., Yi K., Shao J. (2012). Effects of substrate temperatures and deposition rates on properties of aluminum fluoride thin films in deep-ultraviolet region. Appl. Opt..

[5] Bridou F., Cuniot-Ponsard M., Desvignes J.-M., Richter M., Kroth U., Gottwald A. (2010). Experimental determination of optical constants of MgF_2_ and AlF_3_ thin films in the vacuum ultra-violet wavelength region (60–124 nm), and its application to optical designs. Opt. Commun..

[6] Vos M.F.J., Knoops H.C.M., Kessels W.M.M., Mackus A.J.M. (2021). Reaction Mechanisms during Atomic Layer Deposition of AlF3 Using Al(CH_3_)_3_ and SF_6_ Plasma. J. Phys. Chem. C.

[7] De Marcos L.V.R., Wheeler V.D., Batkis M.F., Del Hoyo J.G., Jin E.N., Walton S.G., Wollack E.J., Quijada M.A., Boris D.R. (2023). Passivation of aluminum mirrors with SF_6_- or NF_3_-based plasmas. Opt. Mater. Express.

[8] López-Reyes P., Enríquez E., Crespillo M.L., Marcos L.V.R.-D., Olivares J., Larruquert J.I. (2023). Unveiling the effects of the surface and in-depth nanostructure on the far-UV optical reflectance of thin fluoride multilayer coatings. Appl. Surf. Sci..

[9] Zhou X., Zhu D., Su Y., Wu F. (2024). Cryo-TEM Study of High-Performance Iron Difluoride Cathode Enabled by Low Temperature CVD Carbon Coating. Adv. Funct. Mater..

[10] Fragalà M.E., Toro R.G., Privitera S., Malandrino G. (2011). MOCVD Fabrication of Magnesium Fluoride Films: Effects of Deposition Parameters on Structure and Morphology. Chem. Vap. Depos..

[11] Atosuo E., Mäntymäki M., Pesonen L., Mizohata K., Hatanpää T., Leskelä M., Ritala M. (2023). Atomic layer deposition of CoF_2_, NiF_2_ and HoF_3_ thin films. Dalton Trans..

[12] Messina D.C., Eller B.S., Scowen P.A., Nemanich R.J. (2022). Comparison of AlF_3_ thin films grown by thermal and plasma enhanced atomic layer deposition. J. Vac. Sci. Technol. A.

[13] George S.M. (2010). Atomic Layer Deposition: An Overview. Chem. Rev..

[14] Lee Y., DuMont J.W., Cavanagh A.S., George S.M. (2015). Atomic Layer Deposition of AlF_3_ Using Trimethylaluminum and Hydrogen Fluoride. J. Phys. Chem. C.

[15] Vos M.F.J., Knoops H.C.M., Synowicki R.A., Kessels W.M.M., Mackus A.J.M. (2017). Atomic layer deposition of aluminum fluoride using Al(CH3)3 and SF6 plasma. Appl. Phys. Lett..

[16] Nieminen H.-E., Ritala M. (2022). Reaction mechanism studies on atomic layer deposition process of AlF3. J. Vac. Sci. Technol. A.

[17] Huang Z., Messina D.C., Eller B.S., Koeck F.A., Scowen P.A., Nemanich R.J. (2021). Multilayer ultraviolet reflective coating based on atomic layer deposited aluminum oxide and fluoride. J. Vac. Sci. Technol. A Vac. Surf. Film..

[18] Mackus A.J.M., Heil S.B.S., Langereis E., Knoops H.C.M., van de Sanden M.C.M., Kessels W.M.M. (2010). Optical emission spectroscopy as a tool for studying, optimizing, and monitoring plasma-assisted atomic layer deposition processes. J. Vac. Sci. Technol. A.

[19] Palenius H. (1966). Spectrum of Singly Ionized Fluorine, F ii. J. Opt. Soc. Am..

[20] Kokkoris G., Panagiotopoulos A., Goodyear A., Cooke M., Gogolides E. (2009). A global model for SF6 plasmas coupling reaction kinetics in the gas phase and on the surface of the reactor walls. J. Phys. D Appl. Phys..

[21] Pilvi T., Ritala M., Leskelae M., Bischoff M., Kaiser U., Kaiser N. (2008). Atomic layer deposition process with TiF_4_ as a precursor for depositing metal fluoride thin films. Appl. Opt..

[22] Heil S.B.S., van Hemmen J.L., van de Sanden M.C.M., Kessels W.M.M. (2008). Reaction mechanisms during plasma-assisted atomic layer deposition of metal oxides: A case study for Al_2_O_3_. J. Appl. Phys..

[23] Lee Y., Sun H., Young M.J., George S.M. (2016). Atomic Layer Deposition of Metal Fluorides Using HF-Pyridine as the Fluorine Precursor. Chem. Mater..

[24] Profijt H.B., Kudlacek P., Van De Sanden M.C.M., Kessels W.M.M. (2011). Ion and Photon Surface Interaction during Remote Plasma ALD of Metal Oxides. J. Electrochem. Soc..

[25] Romanova M., Chertopalov S., Dekhtyar Y., Fekete L., Lančok J., Novotný M., Pokorný P., Popov A.I., Sorokins H., Vilken A. (2025). Charge trapping in SiO2 substrate during electron beam deposition of CaF_2_ thin films of different thicknesses. Opt. Mater. X.

[26] Skriabin A., Telekh V., Pavlov A., Pasynkova D., Podlosinskaya A., Novikov P., Zhupanov V., Chesnokov D., Senkov V., Turyanskiy A. (2023). Surface Degradation of Thin-Layer Al/MgF_2_ Mirrors under Exposure to Powerful VUV Radiation. Nanomaterials.

[27] Lee C.-C., Liao B.-H., Liu M.-C. (2008). Developing new manufacturing methods for the improvement of AlF_3_ thin films. Opt. Express.

[28] Moulder J.F., Stickle W.F., Sobol P.E., Bomben K.D. (1992). Handbook of X-Ray Photoelectron Spectroscopy.

[29] Gutiérrez-Luna N., Perea-Abarca B., Espinosa-Yáñez L., Honrado-Benítez C., De Lis T., Rodríguez-de Marcos L.V., Aznárez J.A., Larruquert J.I. (2019). Temperature Dependence of AlF3 Protection on Far-UV Al Mirrors. Coatings.

[30] Taki Y. (2004). Film Structure and Optical Constants of Magnetron-Sputtered Fluoride Films for Deep Ultraviolet Lithography. Vacuum.

[31] Atosuo E., Mäntymäki M., Ritala M. (2025). ALD of Metal Fluorides–Potential Applications and Current State. Adv. Mater. Interfaces.

[32] Chaya B.M., Pattnaik P.K., Narayan K. (2020). Modeling and Analysis of Organic Light Emitting Diode with Thin Film Anti-Reflective Coatings. J. Nanoelectron. Optoelectron..

[33] Kanmaz İ., Demir S., Kiztanir G., Tomakin M., Nevruzoğlu V. (2025). Metal-Organic Frameworks (MOFs) as an Anti-Reflective Coating for Crystalline Silicon Solar Cells. J. Inorg. Organomet. Polym. Mater..

[34] Mäntymäki M., Heikkilä M.J., Puukilainen E., Mizohata K., Marchand B., Räisänen J., Ritala M., Leskelä M. (2015). Atomic Layer Deposition of AlF_3_ Thin Films Using Halide Precursors. Chem. Mater..

[35] Alan U. Atomic Layer Deposition of Aluminum Fluoride for Use in Optical Devices. Ph.D. Dissereation.

[36] Reif J., Knaut M., Killge S., Albert M., Mikolajick T., Bartha J.W. (2022). In situ studies on atomic layer etching of aluminum oxide using sequential reactions with trimethylaluminum and hydrogen fluoride. J. Vac. Sci. Technol. A.

[37] Sales M.G., Boris D.R., Rodriguez De Marcos L.V., Hart J.L., Lang A.C., Albright B.S., Kessler T.J., Wollack E.J., Quijada M.A., Walton S.G. (2025). Passivation Strategies for Far-Ultraviolet Al Mirrors Using Plasma-Based AlF_3_ Processing. Chem. Mater..

[38] Rodriguez De Marcos L.V., Wheeler V.D., Sales M.G., Del Hoyo J.G., Batkis M.F., Lang A.C., Quijada M.A., Wollack E.J., Walton S.G., Boris D.R. (2024). Advances in plasma-based atomic layer processing of AlF_3_ for the passivation of broadband Aluminum mirrors. Adv. Opt. Mech. Technol. Telesc. Instrum. VI.

