# Supplementary material for: Plasma-Enhanced Atomic Layer Deposition of AlF3 Antireflective Coatings via Pulse-Time Control of Fluorine Radical Reactions
Source: Nanomaterials (Basel). 2025 Dec 29;16(1):43. doi: 10.3390/nano16010043 (PMC12788101; doi:10.3390/nano16010043)
Supplement: Supplementary file 1 [file nanomaterials-16-00043-s001.zip › nanomaterials-4044264-supplementary.pdf]

Supporting Information

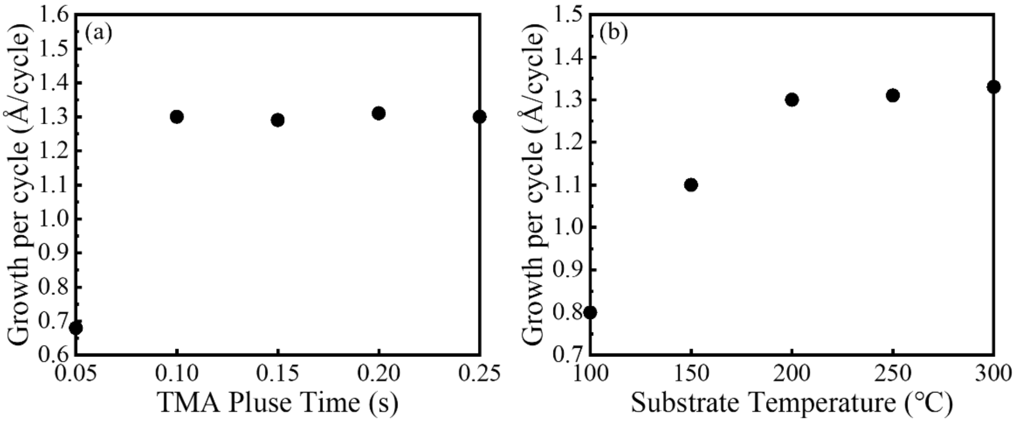

Figure S1. GPC as a function of (a) TMA pulse time and (b) substrate temperature.
